# The role of *SLC26A4* in bony labyrinth development and otoconial mineralization in mouse models

**DOI:** 10.3389/fnmol.2024.1384764

**Published:** 2024-04-29

**Authors:** Taku Ito, Hiroki Watanabe, Keiji Honda, Taro Fujikawa, Ken Kitamura, Takeshi Tsutsumi

**Affiliations:** ^1^Department of Otorhinolaryngology, Tokyo Medical and Dental University, Tokyo, Japan; ^2^Department of Otorhinolaryngology, Chigasaki Chuo Hospital, Kanagawa, Japan

**Keywords:** bony labyrinth, otic capsule, otoconia, micro-CT, incomplete partition type II, *SLC26A4*

## Abstract

Inner ear malformations are predominantly attributed to developmental arrest during the embryonic stage of membranous labyrinth development. Due to the inherent difficulty in clinically assessing the status of the membranous labyrinth, these malformations are diagnosed with radiographic imaging, based on the morphological characteristics of the bony labyrinth. While extensive research has elucidated the intricacies of membranous labyrinth development in mouse models, comprehensive investigations into the developmental trajectory of the bony labyrinth, especially about its calcification process, have been notably lacking. One of the most prominent types of inner ear malformations is known as incomplete partition (IP), characterized by nearly normal external cochlear appearance but pronounced irregularities in the morphology of the modiolus and inter-scalar septa. IP type II (IP-II), also known as Mondini dysplasia, is generally accompanied by an enlargement of the vestibular aqueduct and is primarily attributed to mutations in the *SLC26A4* gene. In the case of IP-II, the modiolus and inter-scalar septa of the cochlear apex are underdeveloped or missing, resulting in the manifestation of a cystic structure on radiographic imaging. In this overview, we not only explore the normal development of the bony labyrinth in mice but also present our observations on otolith mineralization. Furthermore, we investigated the specifics of bony labyrinth and otolith mineralization in *Slc26a4*-deficient mice, which served as an animal model for IP-II. We ensured that these findings promise to provide valuable insights for the establishment of therapeutic interventions, optimal timing, targeted sites, and preventive measures when considering the management of this condition.

## 1 Introduction

**Figure 1:**
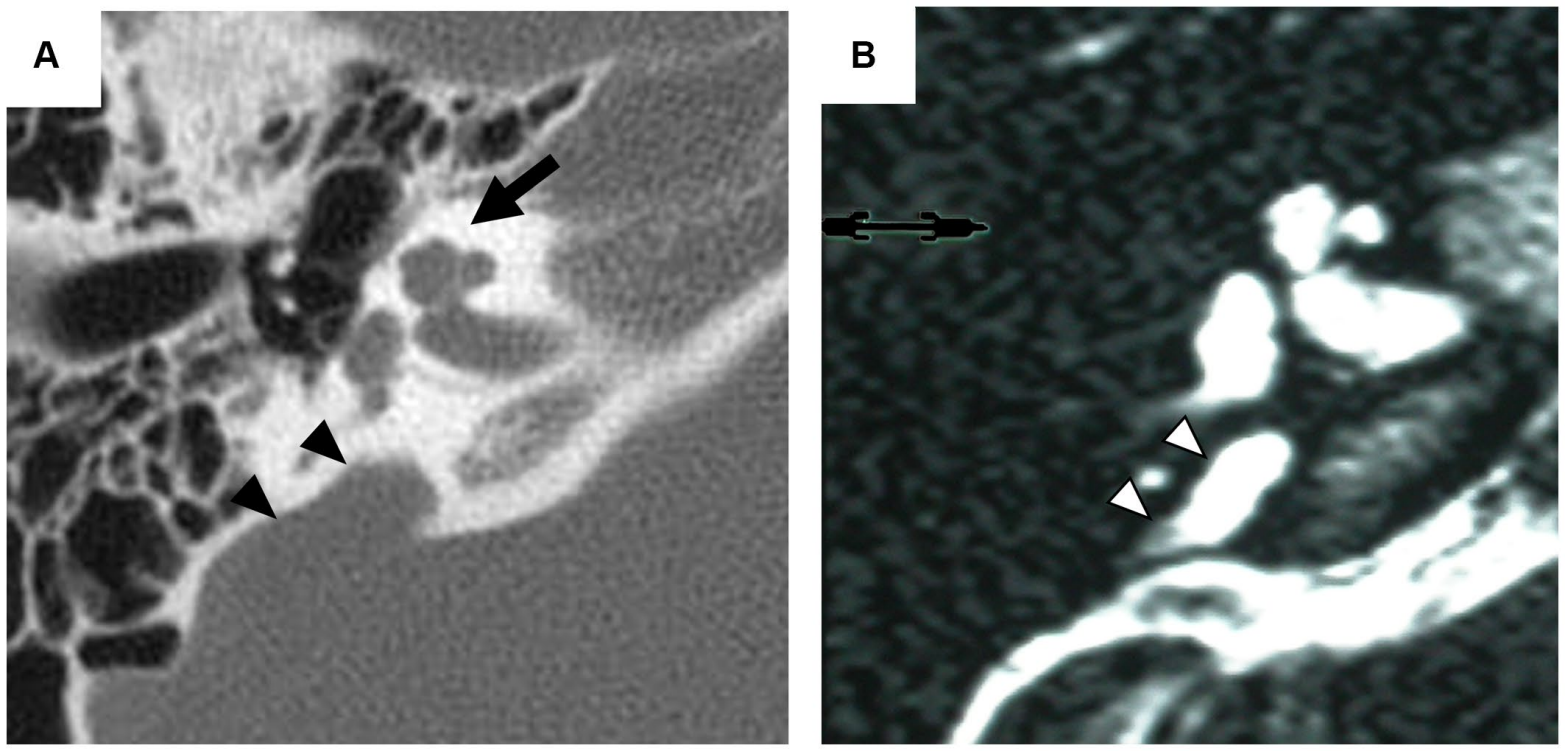
Radiographic imaging of the inner ear in a patient with incomplete partition type II. Axial computed tomography (CT) scan **(A)** exhibits the cystic appearance of the apical turn of the cochlea (arrow) and the enlarged vestibular aqueduct (arrowhead). Axial T2-weighted magnetic resonance (MR) image **(B)** provides an enlarged endolymphatic duct (arrowhead).

Inner ear malformations often arise from developmental delay during the formation of the membranous labyrinth. Clinical confirmation of these anomalies poses challenges due to the difficulty of assessing the state of the membranous labyrinth without post-mortem pathology (Schuknecht et al., 2010). Therefore, classifications primarily rely on the morphology of the bony labyrinth identifiable by computed tomography (CT) or magnetic resonance imaging (MRI) (Jackler et al., 1987; Sennaroglu and Saatci, 2002). One of the most common detectable anomalies, incomplete partition (IP), exhibits normal external cochlear appearance but abnormalities in the modiolus and inter-scalar septa. IPs are classified into various types according to the degree of defects in the modiolus and inter-scalar septa, which are caused by different genetic mutations (Sennaroğlu and Bajin, 2017; Brotto et al., 2021). IP type II (IP-II) lacks the apical part of the modiolus and the corresponding inter-scalar septa are defective, giving the apex of the cochlea a cystic appearance owing to the confluence of the middle and apical turn (Figure 1). IP-II is often associated with genetic mutations, notably those in the *SLC26A4* gene (Ito et al., 2011, 2013; Watanabe et al., 2023).

*SLC26A4* encodes an 86-kDa transmembrane protein named pendrin and plays a crucial role in regulating ion transport in the inner ear (Griffith and Wangemann, 2011). The expression of pendrin in mice is initially observed in the endolymphatic sac at embryonic day 11.5 (E11.5), followed by slightly delayed expression in the stria vascularis of the cochlea and dark cells of the vestibule (Royaux et al., 2003; Kim and Wangemann, 2011). In *Slc26a4*-deficient mice lacking pendrin expression, a hydrops-like expansion of the membranous labyrinth is observed from E14.5 (Kim and Wangemann, 2011). Furthermore, expanded marginal cells are observed in the stria vascularis without scala media expansion, indicative of cellular-level hydropic degeneration (Ito et al., 2015). The *Slc26a4*-deficient mice have also demonstrated abnormalities in otoconial formation, which could lead to impaired vestibular function. Mutations in *SLC26A4* in human are linked to various inner ear anomalies, including IP-II and the enlargement of the vestibular aqueduct (Everett et al., 1997; Campbell et al., 2001). *SLC26A4* is also a causative gene for Pendred syndrome which is associated with a combination of sensorineural hearing loss, vestibular dysfunction, structural abnormalities in the inner ear and thyroid abnormalities (Baldwin et al., 1995; Everett et al., 1997; Li et al., 1998).

Significant advances have been made in understanding the development of the membranous labyrinth and its pathologies associated with SLC26A4 deficiency, as demonstrated by numerous foundational studies (Everett et al., 2001; Royaux et al., 2003; Choi et al., 2011; Ito et al., 2014). A comprehensive account of the development of the mouse inner ear and the origination of its sensory organs has been provided, establishing a baseline for studying the impact of genetic mutations (Morsli et al., 1998). Furthermore, investigations have uncovered the molecular mechanisms behind stereocilia defects and emphasized the critical role of specific genes, such as *Pax2*, in the development of the inner ear (Littlewood Evans and Müller, 2000; Burton et al., 2004). These findings clarify the complex genetic framework essential for the proper formation and functioning of the inner ear, shedding light on the impacts of genetic mutations. However, despite the thorough research into the anomalies of the membranous labyrinth in various animal models, a noticeable research gap exists in studies focused on the bony labyrinth’s anomalies. This underscores the importance of additional research to fully understand the genetic factors’ influence on the development of the ear’s bony structures. The review seeks to bridge this gap by elucidating the normative development of the bony labyrinth in mice and presenting our findings on otoconial mineralization. This comprehensive review offers an evaluation of the bony labyrinth, observed under conditions of minimal artifact interference via non-destructive methods employing micro-CT. Additionally, we examine the calcification of the otic capsule and mineralization of otoconia in a model mouse, specifically focusing on the SLC26A4-deficient mouse, a recognized model for IP-II.

## 2 Development of the murine bony labyrinth

**Figure 2:**
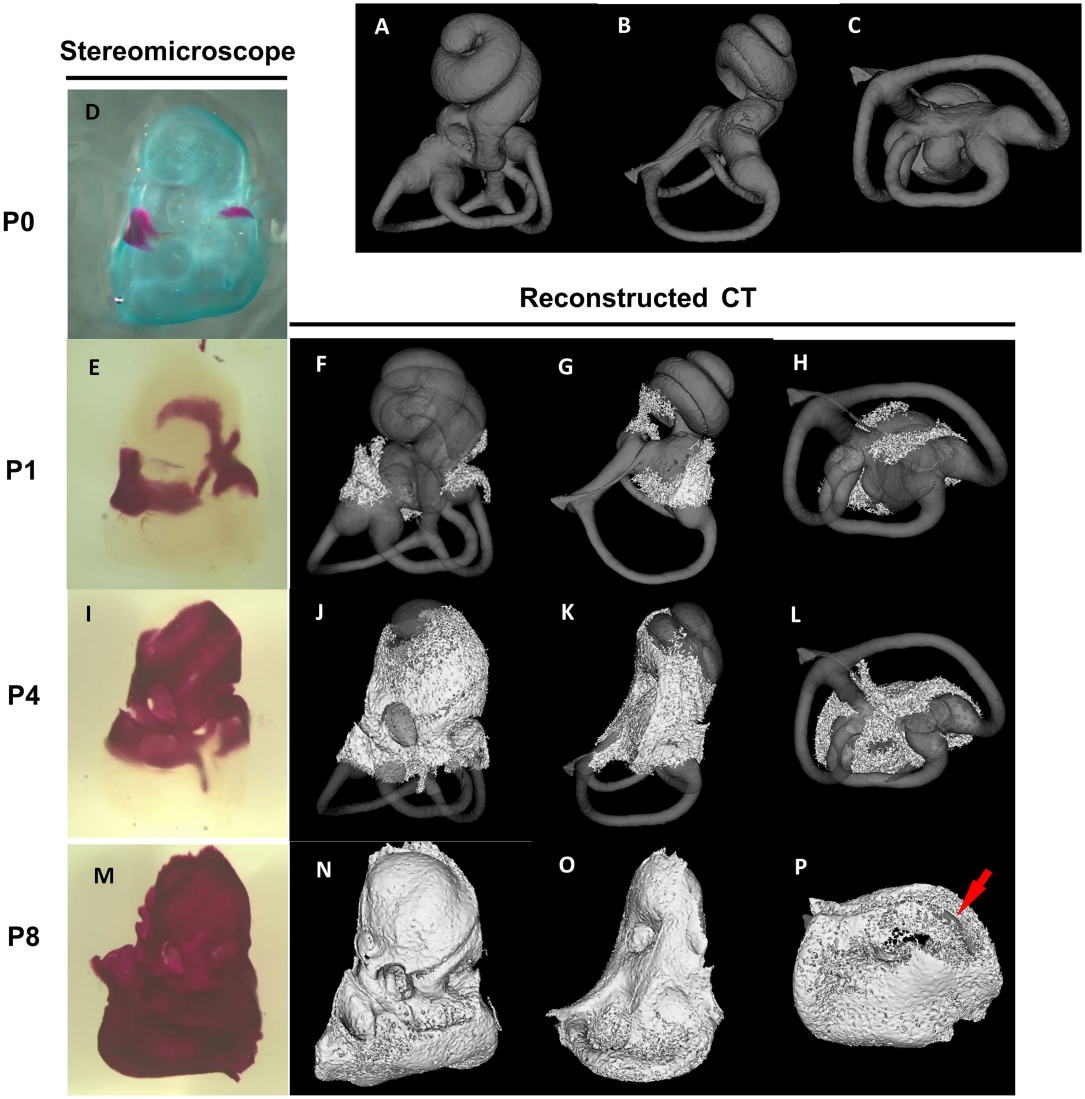
Stereomicroscopic and reconstructed computed tomography appearances of otic capsule ossification. Panels **(A–C)** show the frontal, lateral, and bottom view of a bony labyrinth constructed by the mature otic capsule without ossification at the age of 3–4 months for the superimposed display. The otic capsule ossification starts from two distinct locations by stereomicroscope panel **(D)**. One ossification center is observed around the ampulla of the anterior semicircular canal and utricle **(E–H)**. It then grows toward the saccule and cochlear middle turn **(I–L)**. The other center is identified around the ampulla of the posterior semicircular canal **(E–H)** and then grows to the cochlear basal turn **(I–L)**. The ossification of the semicircular canals is almost completed **(M–P)**, except for the anterior semicircular canal (arrow in **P**).

The differentiation of the membranous labyrinth in the mouse inner ear initiates from the crista of the posterior semicircular canal at E11.5, extending toward the ampulla of the anterior and lateral semicircular canals and the utricle macula (Morsli et al., 1998). Additionally, by the age of E13, a clear differentiation is observed in the formation of the saccule macula and cochlear duct (Morsli et al., 1998). All primordial structures undergo further refinements, approximating their mature shape at E15 (Morsli et al., 1998). The development of the bony labyrinth initiates postnatally from two primary ossification centers near the ampulla of the anterior semicircular canal and the ampulla of the posterior semicircular canal, followed by the cochlea and vestibule (Bai et al., 2023). In the cochlea, external surface ossification progresses from the basal to the apical turn, covering almost all regions with bone tissue by approximately postnatal day 8 (P8) (Bai et al., 2023) (Figure 2). Conversely, in the vestibule system, the external surface ossification process starts near the ampullae, extending toward the utricle, saccule and individual semicircular canals (Bai et al., 2023). The ossification of the anterior semicircular canal culminates as the final stage in this process. By P8, the membranous labyrinth is essentially covered by bone tissue, and subsequent ossification leads to an increase in bone density and thickness across the entire otic capsule (Bai et al., 2023) (Figure 2). The rise in bone density continues approximately until 2–3 months postnatal (Bai et al., 2023). The part of the bony labyrinth covering the utricle and saccule also gradually enlarges within the same period. The external surface ossification process of the otic capsule predominantly proceeds through endochondral ossification (Anson et al., 1948).

However, the internal ossification processes present a distinctive developmental feature. Unlike other areas, ossification of the modiolus and interscalar septum proceeds not through a transition from cartilaginous tissue but through membranous ossification. The ossification of the mouse modiolus starts around the basal turn at P4. From P5 to P6, it rapidly elongated in the apical direction in a spiral manner (Bai et al., 2023). Modiolar ossification spread to the end of the apical turn at P8. The ossification of the interscalar septa initiates laterally from the modiolus and medially from the bony otic capsule at P8, reaching near completion by P15 (Bai et al., 2023).

## 3 Development of the murine otoconial mineralization

**Figure 3:**
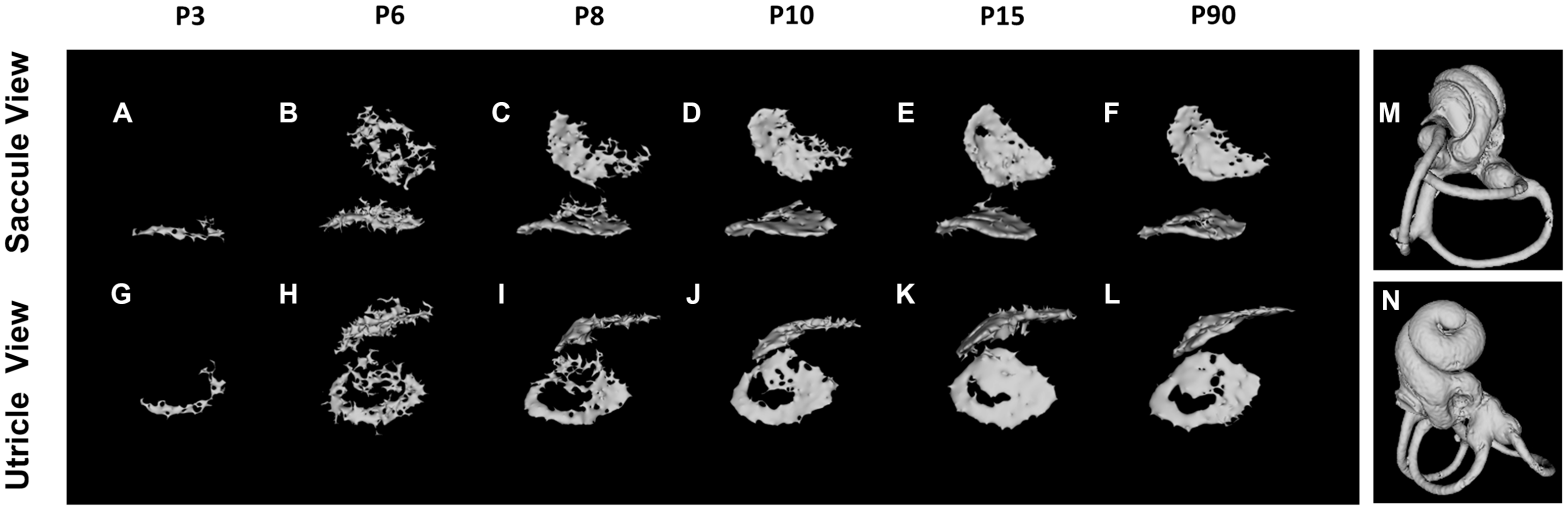
Otoconium mineralization. The utricular otoconia are first detected at P3 by reconstructed micro-computed tomography **(A,G)**, whereas the saccular otoconia are identified at P6 **(B,H)**. The otoconium mineralization gradually develops between P6 and P10, and the calcium depositions on the otoconia on the utricle and saccule are almost completed at P15 **(E,K)**, the computed tomography images of which are almost identical to those at P3–4 Mo **(F,L)**. The orientations of Saccule and Utricle view are designated as **(M,N)**.

The otoconia, small calcified structures in the vertebrate inner ear, are composed of calcium carbonate and a protein matrix (Gil-Loyzaya, 1985; Pote, 1986). These crystalline particles are pivotal in detecting gravity and linear acceleration, thereby playing an integral role in balance and spatial orientation. The mineralization of otoconia is critical for the vestibular function, as it allows organisms to sense changes in head position and movement. Disruptions in otoconial mineralization can result in vestibular disorders, impacting equilibrium and spatial awareness. Notably, otoconial mineralization begins prenatally (Preston, 1975), with micro-CT enabling morphological observation at P3 (Ito et al., 2020; Bai et al., 2023). The mineralization process varies between saccular and utricular otoconia but is largely complete by P15 (Figure 3). The morphology and degree of mineralization of otoconia remain consistent up to 2–3 months postnatal (Bai et al., 2023).

## 4 *Slc26a4*-deficient mice

**Figure 4:**
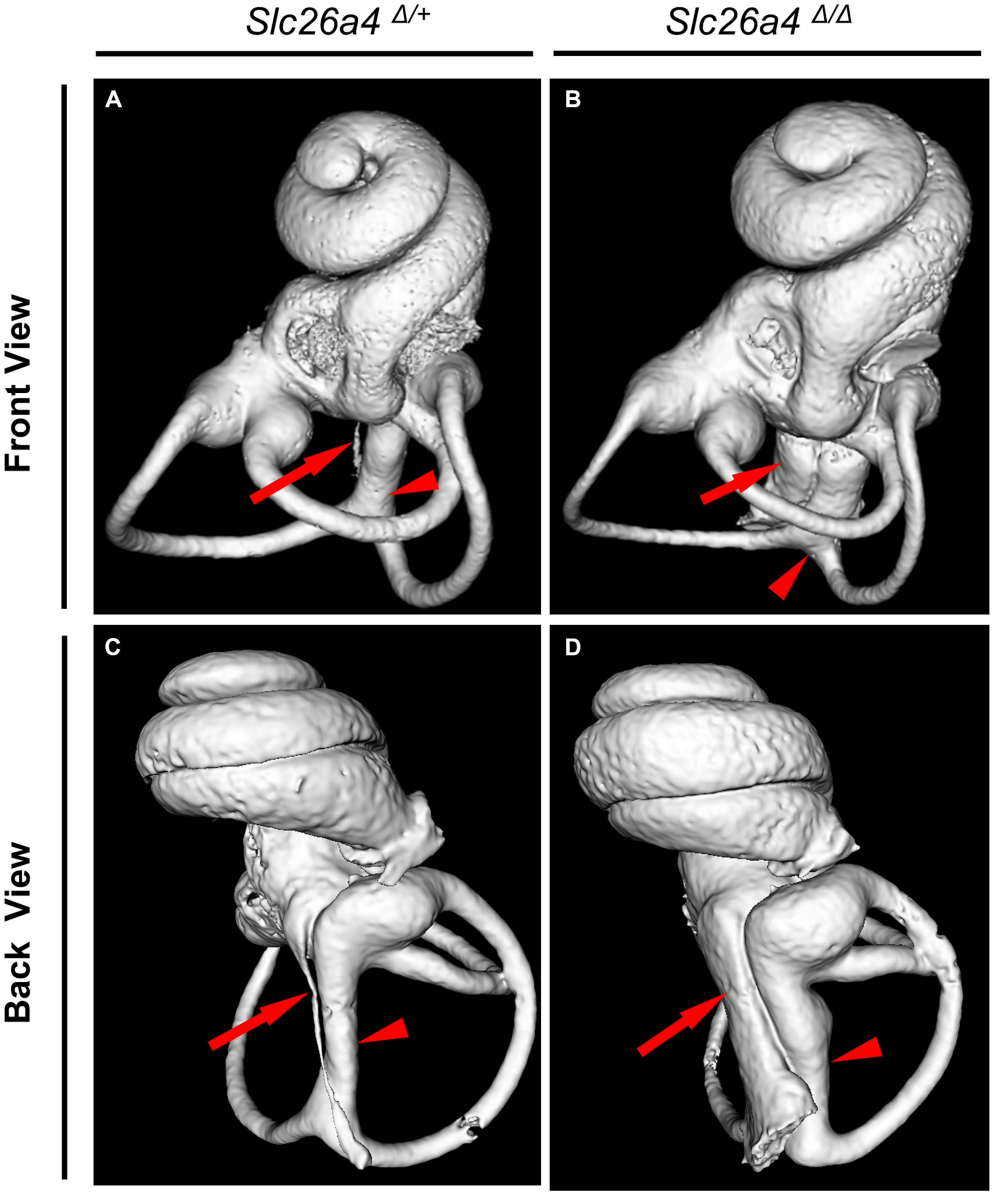
Bony labyrinth appearances. Panels **(A,B)** show the front views, and **(C,D)** show the back views of the bony labyrinth reconstructed by computed tomography. The bony labyrinths of common crus (arrowheads) and endolymphatic duct (arrows) are enlarged in the ear of *Slc26a4 ^Δ Δ^* **(B,D)**, compared with in the ear of *Slc26a4 ^Δ/+^* **(A,C)**.

The *Slc26a4*-deficient mouse model, which lacks functional expression of the *Slc26a4* gene, serves as a crucial tool in understanding the role of the *SLC26A4* gene in the inner ear and other human tissues (Griffith and Wangemann, 2011; Ito et al., 2014). This gene is responsible for encoding pendrin, a protein essential for the normal functioning of the inner ear (Everett et al., 2001; Royaux et al., 2003). In the absence of *Slc26a4* expression, these mice exhibit a range of auditory and vestibular abnormalities, including severe sensorineural hearing loss, vestibular deficits, acidic endolymph, and significant enlargement of endolymphatic spaces in the inner ear (Everett et al., 2001; Royaux et al., 2003; Choi et al., 2011; Ito et al., 2014, 2015; Nishio et al., 2016; Watanabe et al., 2023) (Figure 4). Notably, abnormal expansion of the scala media and endolymphatic sac in these mice begins as early as E14.5 (Everett et al., 2001; Kim and Wangemann, 2011). This early onset results in congenital enlargement of the vestibular aqueducts, mirroring the radiographic deformity observed in humans with Pendred syndrome (IP-II anomaly) (Ito et al., 2021) (Figure 1).

**Figure 5:**
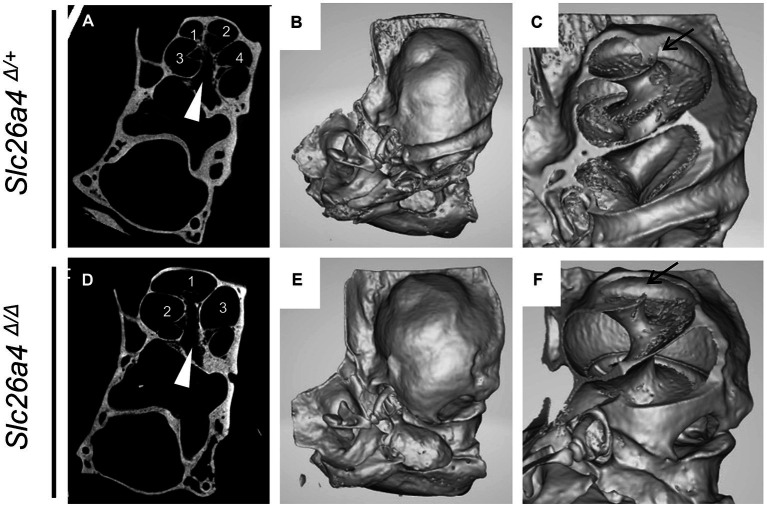
Representative gross and cross-sectional images of otic capsules. Mid-modiolar planes reveal four cross sections (1–4) of the cochlear duct separated by bony septa in *Slc26a4^Δ/+^* mice **(A,C)** but only three cross sections (1–3) in *Slc26a4^Δ/Δ^* mice **(D,F)**. Apical turns appeared cystic without bony septa in this plane **(D)**. Three-dimensional construction **(B,C,E,F)** failed to detect bony septa in the apical end (arrow in **F**). Arrowheads indicate the center of modiolus.

In *Slc26a4*-deficient mice, the otolith organs exhibit a marked lack of regular otoconia, alongside the occasional presence of abnormally large, or giant, otoconia (Everett et al., 2001; Ito et al., 2020; Watanabe et al., 2023) (Figure 5). Otoconial formation in these mice starts around E14.5, with a peak in mineralization rates occurring between E15 and E16 (Anniko, 1980; Anniko et al., 1987; Lowenstam and Weiner, 1989). It is noteworthy that otoconia undergoes a continuous turnover process. In this process, supporting cells in the otolith organs act as the site of generation, while dark cells function as the absorption site. The formation of giant otoconia is hypothesized to stem from the dissolution and reaggregation of smaller otoconia, likely due to biochemical abnormalities in the endolymph (Dror et al., 2010).

## 5 Enlarged bony labyrinth without *Slc26a4* expression

In the cochlear region of *Slc26a4*-deficient mice, multiplanar reconstructed images obtained via micro-CT have disclosed developmental delays and arrested ossification of the interscalar septa at the apical turn (Wangemann et al., 2009; Ito et al., 2021). This anomaly renders the apical turn of the cochlea cyst-like in appearance, creating the illusion of a reduced number of cochlear turns at first glance (Ito et al., 2021) (Figure 5). However, when comparing the *Slc26a4*-deficient mice to controls, the rotation angles from the ductus reunions to the apical end around the modiolus exhibit similarity (Ito et al., 2021) (Figures 4A,B). Nonetheless, a notable difference is observed in the modiolus width, which is thinner in *Slc26a4*-deficient mice. This observation underscores the essence of mouse IP-II anomaly, characterized primarily by modiolar hypoplasia and the loss or deossification of the interscalar septum.

In the vestibular region of these mice, an increase in the size of the bony labyrinth surrounding the utricle and saccule is evident (Ito et al., 2020). Additionally, the dimensions of the vestibular aqueduct, common crus, and anterior semicircular canal are also enlarged (Watanabe et al., 2023). In addition, the closer the structure is to the endolymphatic sac and duct, the more pronounced the impairment in the vestibular apparatus, suggesting that the impaired absorption of endolymphatic fluid in the endolymphatic sac is likely the primary contributing factor.

## 6 Abnormal otoconial formation without *Slc26a4* expression

**Figure 6:**
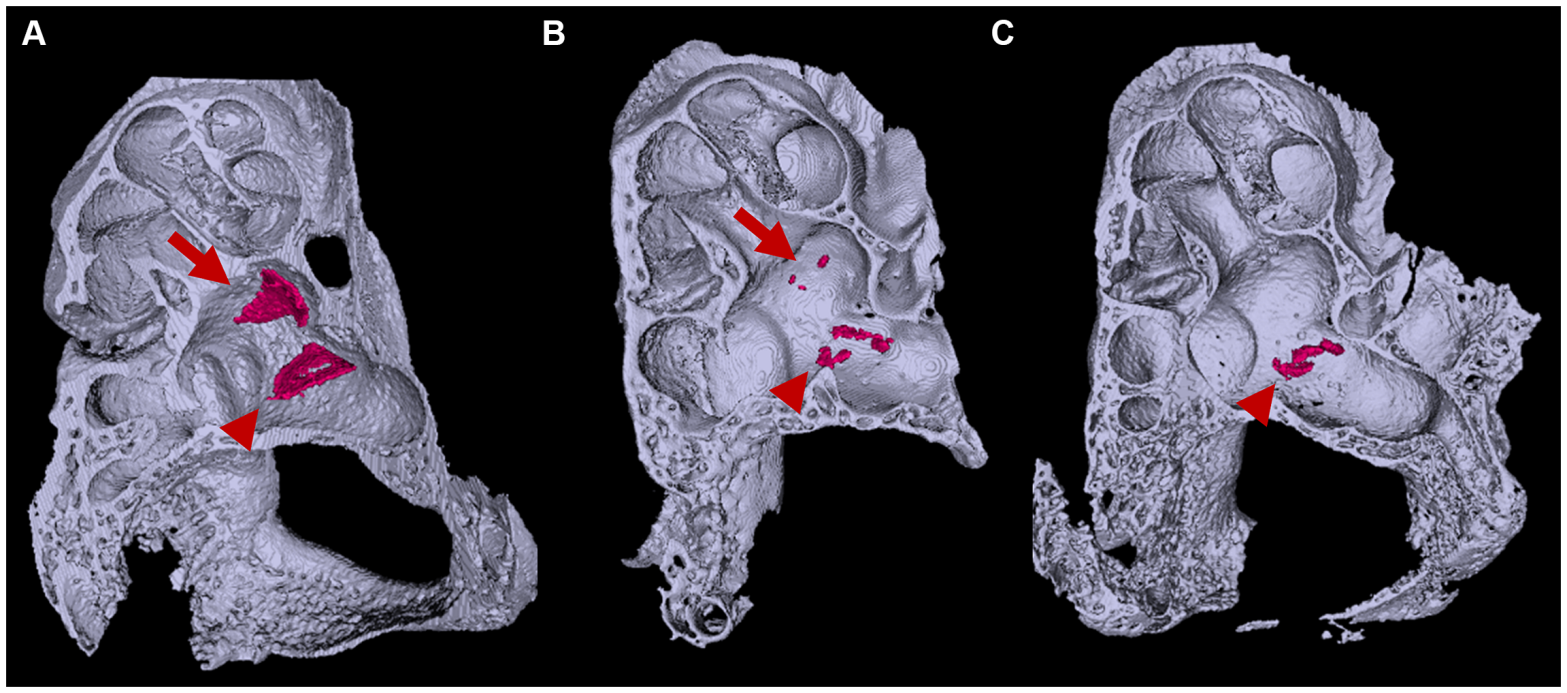
Three-dimensional appearance of otoconia in the otic capsule. Three-dimensional appearances of **(A)** *Slc26a4^Δ/+^* and **(B,C)** *Slc26a4^Δ/Δ^* otic capsules at P8. Arrows and arrowheads indicate otoconia aggregates in the saccule and utricle maculae, respectively. The surface-rendering technique creates triangular aggregates of the otoconia (red) in the saccule and utricle maculae in *Slc26a4^Δ/+^* mice ears. By contrast, several to a dozen small aggregates are observed in the utricle in *Slc26a4^Δ/Δ^* mouse ears, corresponding to giant otoconia. The otoconium in the saccule are few or absent **(B,C)**.

In the *Slc26a4*-deficient mouse model, a distinctive pattern of otoconial development is observed. By P8, several to approximately 20 giant otoconia are found within the utricle, whereas the saccule typically exhibits a complete absence or contains only 1 or 2 otoconia (Ito et al., 2020; Watanabe et al., 2023) (Figure 6). As the mice age, these otoconia not only enlarge further but also decrease in number (Ito et al., 2020). Under normal circumstances, otoconia, secured by a filamentous matrix, are confined above the sensory epithelium of the saccule and utricle, preventing their displacement into other endolymphatic spaces. However, in the *Slc26a4*-deficient mice, giant otoconia demonstrate notable instability on the otoconial membrane, often dislodging during experimental processing. This instability is evidenced by the frequent observation of abnormal, ectopic otoconia in the semicircular canals and their cristae in histological sections (Dror et al., 2010), indicative of a disruption in normal *Slc26a4* expression.

In contrast, micro-CT imaging suggests that each otoconium remains relatively anchored to its original position on the otolithic membrane, with no ectopic otoconia detected in the semicircular canals (Watanabe et al., 2023). This discrepancy between previous histological findings and micro-CT data calls for further investigation. The differing outcomes raise questions about whether these inconsistencies are due to the inherent resolution limitations of micro-CT or result from potential artifacts introduced during the histological sectioning process.

## 7 Correlating otoconial dynamics in murine models with vestibular dysfunction in Pendred syndrome

The prevalence of equilibrium disorders in Pendred syndrome, characterized by fluctuating and progressive hearing loss along with episodes of vertigo and imbalance, represents a significant clinical challenge. Our investigations utilizing *Slc26a4*-deficient mice have revealed that the principal contributors to vestibular dysfunction are not disturbances in vestibular hair cells, but rather the significant enlargement and inherent instability of otoconia, accompanied by their temporal changes (Watanabe et al., 2023). These findings in murine models provide new insights into the pathomechanisms potentially active in human Pendred syndrome. We hypothesize that alterations in the size and spatial arrangement of otoconia are crucial for the onset of vestibular impairments, underscored by the dynamic nature of otoconial behavior observed. This insight significantly elevates the understanding of the pathophysiology of vestibular disorders, both in experimental contexts and clinical settings. Moreover, the absence of a direct correlation between the size of the vestibular aqueduct and the severity of symptoms, together with the variability in vestibular dysfunction among individuals, highlights the complexity of this syndrome (Schuknecht et al., 2010). Our study provides essential evidence that supports the hypothesis that the pathophysiological alterations observed in Pendred syndrome, both in humans and mouse models, are closely linked to disturbances in otoconial dynamics. This could lead to the development of specific interventions to manage vestibular symptoms in affected individuals.

## 8 Management of auditory and vestibular impairments associated with IP-II and *SLC26A4* mutations

To date, research has established that pendrin expression in the endolymphatic sac is critical for the acquisition of auditory and vestibular functions, whereas its expression in other regions appears less essential (Li et al., 2013). A reduction in pendrin levels within the stria vascularis has been linked to fluctuations in auditory function (Nishio et al., 2016). Additionally, while cochlear hair cells are prone to early impairment and eventual loss, vestibular hair cells generally preserve their morphological integrity for several months postnatally (Dror et al., 2010; Takeda et al., 2019; Watanabe et al., 2023). These observations collectively suggest that future therapeutic strategies for auditory-vestibular impairments should prioritize early developmental interventions targeting the endolymphatic sac to acquire auditory function. Post-acquisition, the focus should shift to preserving hearing by addressing the stria vascularis. Furthermore, for the management of balance disorders, there is a pressing need to develop treatments that stabilize the otolith organs and their morphology. Currently, no fundamental treatment exists for balance disorders associated with Pendred syndrome, although hearing impairments are addressed with cochlear implants (Yan et al., 2013; Wu et al., 2015). The exploration of pathogenesis in balance disorders using the mouse model may pave the way for innovative treatment approaches.

## Author contributions

TI: Conceptualization, Funding acquisition, Project administration, Resources, Supervision, Visualization, Writing – original draft, Writing – review & editing. HW: Validation, Writing – review & editing. KH: Validation, Writing – review & editing. TF: Validation, Writing – review & editing. KK: Validation, Writing – review & editing. TT: Validation, Writing – review & editing.
